# ATMPs prepared under hospital exemption: European Blood Alliance position paper

**DOI:** 10.1002/hem3.70215

**Published:** 2025-09-23

**Authors:** Dragoslav Domanović, Marc Turner, Christof Jungbauer, Bernardo Rodrigues, Johanna Nystedt, Primož Rožman, Monique Debattista, Tengyu Wang, Einar Klæboe Kristoffersen, Kalinga Perera, Urban Švajger, Lilian Hook, Marjolaine Jacques, Ana Paula Sousa, Marten Hansen, Peter O'Leary, Pierre Tiberghien, European Blood Alliance Ad Hoc ATMPs Group

**Affiliations:** ^1^ European Blood Alliance Brussels Belgium; ^2^ Scottish National Blood Transfusion Service, The Jack Copland Centre Edinburgh UK; ^3^ Austrian Red Cross, Blood Service for Vienna, Lower Austria and Burgenland Vienna Austria; ^4^ Department of Transfusion Medicine Paracelsus Medical University Hospital Salzburg Austria; ^5^ Finnish Red Cross Blood Service Vantaa Finland; ^6^ Slovenian Institute for Transfusion Medicine Ljubljana Slovenia; ^7^ National Blood Transfusion Service Pietà Malta; ^8^ Karolinska University Hospital Stockholm Sweden; ^9^ Laboratory Medicine and Pathology, Department of Immunology and Transfusion Medicine Haukeland University Hospital Bergen Norway; ^10^ Welsh Blood Service Llantrisant UK; ^11^ NHS Blood and Transplant Bristol UK; ^12^ French Blood Establishment (EFS) La Plaine Saint‐Denis France; ^13^ Portuguese Institute of Blood and Transplantation Lisboa Portugal; ^14^ Laboratory for Cell Therapy and Amsterdam UMC, Department of Research, Sanquin Blood Supply Foundation University of Amsterdam Amsterdam The Netherlands

In the European Union, advanced therapy medicinal products (ATMPs)—including somatic cell therapies, gene therapies, and tissue‐engineered products—are generally subject to centralized marketing authorization by the European Medicines Agency (EMA). However, the ATMP Regulation (EC) No 1394/2007 also allows their production and use within the country under the hospital exemption (HE) clause, provided that specific criteria are met and approved by the National Competent Authorities.^1^

Since the ATMP Regulation was introduced in 2007, the ATMP field has experienced significant scientific and technological advancements, whereas the regulatory framework remains largely unchanged and appears overly restrictive, potentially hindering the development and availability of these innovative therapies. Although many health conditions may benefit from ATMP therapies, the high development and manufacturing costs, which are also driven by regulatory requirements, often discourage commercial investment, especially for rare diseases or niche applications. In some instances, marketing authorizations have been withdrawn or not renewed, mainly for commercial reasons rather than due to clinical issues.^2^

In contrast, HE is widely acknowledged as a valuable tool, enabling access to innovative and often life‐saving treatments in cases of unmet clinical need. However, the current HE provisions—restricting production to the same Member State, requiring nonroutine preparation within the hospital, and assigning responsibility to a single practitioner—are ill‐suited to today's clinical and manufacturing realities. These limitations reduce the scalability and broader applicability of ATMP production, thereby affecting supply. Furthermore, disparities in how Member States interpret and implement HE have resulted in inconsistent quality, safety, and efficacy standards across the EU.^3^ Additionally, the EU lacks a system for monitoring health outcomes in patients treated with ATMPs through HE. Developing such a system is crucial to ensure transparency in quality and safety, generate scientific knowledge, enhance healthcare efficiency, and raise stakeholder awareness.

A further challenge lies in the ambiguous regulatory classification boundary between ATMPs and substances of human origin (SoHO), which results in legal uncertainty over applicable frameworks. The European Commission's 2023 proposal to revise the Medicinal Products Directive includes provisions for improved data collection and annual reviews of ATMPs produced under HE, with public reporting through an EMA‐managed repository. Recital 18 of the proposal also suggests the potential for an adapted framework for less complex ATMPs manufactured under HE.^4^

Although industry associations have generally supported tighter restrictions on the use of HE,^5^ the European Blood Alliance (EBA), alongside multiple scientific societies and professional organizations, has expressed strong support for maintaining and strengthening this exemption.^6^ The EBA comprises 29 national blood services that represent the majority of public blood collection in the EU, EFTA, and the United Kingdom.^7^ Besides core activities, EBA members participate in developing and producing cell‐based ATMPs. In response to the differing views within the industry, the EBA has intensified its focus on ATMPs by launching a survey among its members regarding their HE‐related activities. Based on the survey results, the EBA convened a working group of experts to develop a position aimed at strengthening the HE framework.

## EBA POSITION

The EBA strongly advocates for expanding, not limiting, the use of HE for ATMPs. Instead of being an exceptional measure, HE should become a harmonized regular approach for producing ATMPs, including those with marketing authorization that are not available nationally. An EU system for monitoring outcomes in treated patients with ATMPs under HE should be established. The EBA also suggests modifying the ATMP framework to include the new elements in the SoHO regulation and reassessing the classification of products as either SoHO products or ATMPs.

The SoHO regulation establishes more stringent standards for SoHO products intended for human use.^8^ Their regulatory approval necessitates strong evidence of safety, quality, and efficacy, along with outcome monitoring and benefit‐risk assessments. These requirements could serve as a basis for reclassifying lower risk cell‐based ATMPs as SoHO products under specific conditions. The development and manufacturing of new ATMPs must continue to be subject to strict regulatory oversight.

The EBA recognizes the essential role of non‐profit SoHO establishments and hospitals in the development and production of cell‐based ATMPs under HE. According to the EBA 2024 survey (manuscript in preparation), blood establishments in many EU countries are actively engaged in ATMP activities within the HE framework. Their involvement reflects the longstanding synergy between SoHOs and many ATMPs. Blood, tissues, and cells often serve as critical starting materials for ATMPs. Public‐sector establishments have played a leading role globally in the development of hematopoietic stem cell transplantation, an affordable and curative treatment for several disorders. Although hematopoietic grafts are not classified as ATMPs, they share multiple characteristics with them. Notably, the success of stem cell transplantation over the past five decades has relied on principles analogous to those underpinning HE.

Moreover, SoHO establishments often provide the trained personnel, infrastructure, equipment, and storage capacity required for the development and production of ATMPs.^9^

Over time, it has become increasingly evident that ATMPs produced under HE share key characteristics with SoHO preparations:

- *Compliance with quality, safety, and efficacy standards* is required for both ATMPs and SoHOs.
- *Individual tailoring* is common in both fields: ATMPs are often personalized for the patient, just as blood components and stem cells are matched by blood group and/or human leukocyte antigen type.
- *Validated manufacturing methods* must be employed in both domains.
- *Oversight by national competent authorities* is mandated for both ATMPs under HE and for SoHO‐based products.

These shared features should be acknowledged by all relevant stakeholders, particularly regulators and policymakers.

The EBA strongly believes that expanding the concept of HE and strengthening the role of public SoHO establishments in the development and production of high‐quality and safe ATMPs will increase the number of manufacturing centers, reduce production costs, and most importantly, enhance patient access to affordable and innovative therapies.

## AUTHOR CONTRIBUTIONS

**All members of the EBA ATMPs Group**: Conceptualization (equal); writing—review and editing (equal). **Dragoslav Domanović**: Conceptualization (lead); writing—original draft (lead); writing—review and editing (equal).

## CONFLICT OF INTEREST STATEMENT

The authors declare no conflicts of interest.

## FUNDING

This research received no funding.

## Data Availability

The data that support the findings of this study are available from the corresponding author upon reasonable request.
